# Does the Vehicle Matter? Real-World Evidence on Adherence to Topical Treatment in Psoriasis

**DOI:** 10.3390/pharmaceutics13101539

**Published:** 2021-09-23

**Authors:** Ana Teixeira, Maribel Teixeira, Vera Almeida, Rita Gaio, Tiago Torres, Sofia Magina, Cátia Cunha, José M. Sousa Lobo, Isabel F. Almeida

**Affiliations:** 1 Instituto de Investigação e Formação Avançada em Ciências e Tecnologias da Saúde (IINFACTS), Instituto Universitário de Ciências da Saúde, CESPU, 4585-116 Gandra, Portugal; ana.teixeira@iucs.cespu.pt (A.T.); vera.almeida@iucs.cespu.pt (V.A.); 2 Laboratório de Tecnologia Farmacêutica, MedTech, UCIBIO-REQUIMTE, Departamento de Ciências do Medicamento, Faculdade de Farmácia, Universidade do Porto, 4050-313 Porto, Portugal; slobo@ff.up.pt; 3 Centro de Matemática, Departamento de Matemática, Faculdade de Ciências, Universidade do Porto, 4169-007 Porto, Portugal; argaio@fc.up.pt; 4 Serviço de Dermatologia, Centro Hospitalar do Porto, Hospital de Santo António, 4099-001 Porto, Portugal; tdtorres@icbas.up.pt; 5 Serviço de Dermatologia, Centro Hospitalar de São João, Departamento de Farmacologia e Terapêutica, Faculdade de Medicina, Universidade do Porto, 4200-319 Porto, Portugal; smagina@med.up.pt; 6 Serviços Farmacêuticos, Centro Hospitalar do Tâmega e Sousa, 4564-007 Penafiel, Portugal; catia_cunha_95@hotmail.com

**Keywords:** adherence, psoriasis, topical treatment, vehicle, mechanical properties

## Abstract

The influence of the vehicle in topical treatment adherence remains to be elucidated. The aim of this study is to analyze the influence of the pharmaceutical dosage form on adherence to topical treatment in psoriasis patients, taking into consideration the mechanical features. The adherence was evaluated in a sample of 102 psoriasis patients, followed for approximately 45 days. Adherence was calculated with a new combined methodology using a log and medication weights. The effect of the group formulation was evaluated using logistic regression models. A complex effect of the vehicle on adherence was found, mediated by the affected area. The adherence was significantly higher for patients applying gels and creams than for those using ointments, whenever the body area affected was extensive. The opposite was found when the affected area was small. Mechanical properties can partially explain the findings since gels and creams may be easier to apply. Patient beliefs and preferences regarding vehicles and their sensory attributes might also explain the results. It is noteworthy that adherence was strikingly low, with more than 75% non-adherent patients. This real-world evidence provides an insight for pharmaceutical industries and guidance for treatment prescription by physicians aiming to address the public health emergency of treatment non-adherence.

## 1. Introduction

Psoriasis is a chronic skin disease with high psychosocial impact [1,2] that is estimated to affect 2% of the world’s population but varies according to regions [3]. Most patients with mild or moderate plaque psoriasis can be managed with topical therapy [4,5]. Topical treatment of psoriasis includes the use of corticosteroids, analogs of vitamin D, tars, anthralin and keratolytics [6]. Although different topical therapeutic approaches are available, low adherence rates can make them inefficient [7,8]. Adherence to topical treatments in psoriasis patients is reported to be low (in the range of 39% to 73%) [8,9,10].

Medication non-adherence is a prevalent health care problem and non-adherence to therapy for chronic diseases is estimated to be around 50%. Non-adherence increases treatment costs associated with poor outcomes and economic costs associated with, for instance, medicine wastage. In Europe, non-adherence is associated with almost 200,000 deaths and EUR 80–125 billion of potentially preventable direct and indirect costs [11]. Improving health outcomes and health system efficiency can be achieved through increased medication adherence [12].

The effectiveness, tolerability and safety of drugs included in topical medicines may vary according to the vehicle (liquid) or base (semi-solid) used, forming different pharmaceutical dosage forms (e.g., ointments, creams, solutions, gels, foams, sprays, shampoos, oils and lotions). In this manuscript, the term vehicle is applied to either liquid or semi-solid formulations [13]. The clinical efficacy of topical treatments for psoriasis is related to treatment adherence, which in turn is associated with vehicle characteristics such as rheological and textural properties, which are dependent both on the excipients and the structure of the matrix [14]. Patient preferences for topical psoriasis treatments have been studied by several authors [15,16,17,18,19,20,21,22], but the objective influence of the topical vehicle and its characteristics on adherence has been scarcely and incompletely addressed. For example, Lambert et al. [16] reported the adherence behavior of patients using calcipotriol/betamethasone dipropionate gel vs. ointment formulations. In this study, however, adherence was evaluated using a self-report questionnaire which did not allow a quantitative measurement, and only two vehicles were tested.

To clarify the importance of the vehicle in psoriasis treatment, besides information about patients’ preferences, real-world evidence studies on adherence and disease outcomes are essential. Topical treatment adherence assessment is a complex issue. None of the published single-measurement methods allow for the accurate assessment of adherence, and for this reason, the use of a combination of methods, including self-report questionnaires, logs and medication weight measurements, has been proposed for a more comprehensive approach [23,24].

Our group has recently characterized the rheological and textural properties of 13 topical medicines for psoriasis [14]. This information is analyzed herein with regard to the influence of the vehicle and its mechanical properties on adherence to topical treatment evaluated via a combination of measurement methods, in a sample of psoriasis patients using a variety of topical formulations as prescribed by the physicians the patients consulted.

## 2. Materials and Methods

This paper is focused on the objective evaluation of the influence of the vehicle on adherence to topical treatment in psoriasis, assessed in a set of 102 patients.

### 2.1. Participants

Patients were selected according to the following inclusion criteria: adults (over 18 years old) with a clinical diagnosis of psoriasis, treated exclusively with topical medicines and with a physician’s prescription for psoriasis treatment. The exclusion condition was patients treated with phototherapy or systemic therapy during the 45 days of the study. All participants signed an informed consent agreement.

### 2.2. Design and Procedures

The adherence to the topical treatment prescribed by the patient’s physician was evaluated in a sample of 102 psoriasis patients, over approximately 45 days. Approval by the Ethics Committees of the following academic and governmental institutions and hospitals was obtained: University of Porto, IUCS (Instituto Universitário de Ciências da Saúde); ARS (Administração Regional de Saúde) Norte, reference number 70, study T421; Hospital de São João; Hospital de Santo António; CNPD (Comissão Nacional de Proteção de Dados), process number 1463/2014, authorization 5343/2014.

#### 2.2.1. Sociodemographic and Clinical Questionnaire

A sociodemographic and clinical questionnaire (constructed specifically for psoriasis patients) was used. Variables related to topical treatment were evaluated for the purpose of this study.

#### 2.2.2. Body Area and Severity Assessment

SAPASI-PT is the Portuguese version of SAPASI and was previously developed and validated by our group [25]. This instrument is a simplified and self-administered version of the Psoriasis Area and Severity Index (PASI) [26,27,28,29].

In this instrument, patients are asked to shade the location of their psoriasis lesions on the front and back of a human figure and to indicate their perception of three characteristics of the lesions: color, thickness and scaliness, based on a visual analogic scale (VAS). To score the instrument, the investigator scores the affected area for each of the following four areas: head, upper extremities, trunk and lower extremities. With this instrument, the affected body area can be determined. The results of the body area affected were dichotomized, according to the median, into “extensive area” (patients with an affected area higher than the median value) and “small area” (patients with an affected area lower than the median value).

#### 2.2.3. Adherence Measurement

Several methods have been used to measure adherence to topical treatments, but no single method allows an accurate and complete assessment of adherence [23]. For a more comprehensive assessment, a combination of different methodologies was used in this work, which represents a new methodological approach. The frequency of use and duration of treatment were analyzed using a log, and the dose (amount used each time) was calculated by weighing the medication.

##### Medication Log

Patients were asked to fill in a medication log showing the number of daily applications of the topical treatment. Adherence (Adherence L) was then calculated as the ratio between the number of applications and the theoretical number of applications, according to the posology and the duration of treatment.

##### Medication Weight

The medicine packages were weighed at the beginning and the end of the study (45 days). Evaluation of adherence by medication weight (Adherence W) was calculated using Equation (1)—Medication weight adherence (%):

Adherence W (%) = *Wu*/*Wex* × 100 (1)

where: *Wu* = medication weight used per application = (weight dispensed–weight returned)/number of applications registered in the medication log; *Wex* = expected medication weight per application = 0.25 g × % *BSA*; *BSA* = body surface area. A value of 0.25 g corresponds roughly to the amount needed for each application on an area corresponding to that of the palm of the hand (1% of *BSA*) [30,31,32]. Values of *BSA* were obtained using SAPASI-PT.

##### Adherence Questionnaire

A questionnaire (QATOP) was also used to collect data related to reasons for non-adherence and treatment-associated variables. QATOP was previously constructed by the authors, evaluated by a panel of experts comprising dermatologists, pharmacists, psychologists and statisticians, and validated regarding content validity [33].

##### Adherence Calculation

Adherence was calculated from the average of the adherence evaluated by the medication log and the medication weight reporting the mass used per application (Adherence Combo). When values were higher than 100%, the result was subtracted from 200 to avoid overestimation of the mean adherence. This means, for example, that 120% is converted to 80%.

To study the effect of the vehicle on adherence to topical treatment, the sample was divided based on the calculated adherence values (without conversion) into two groups: “non-adherent” (with an adherence rate of <80% or >120%) and “adherent” (those who adhered to the prescribed treatment with an adherence rate of 80–120%).

#### 2.2.4. Evaluation of the Influence of the Vehicle on Adherence

The mechanical properties, namely flow behavior and textural properties, of 13 topical formulations commercially available for psoriasis treatment (eight ointments, four creams and one gel) were analyzed in a previous study [14]. A power-law model was fitted to the flow results, and its parameters, consistency coefficient (K) and power-law index (n), were calculated for all formulations. In brief, all formulations presented shear-thinning behavior with power-law indexes (n) lower than 1. The consistency coefficient was higher for ointments, while the oleogel presented less pronounced shear-thinning behavior. Regarding textural analysis, ointments presented higher adhesiveness and higher firmness and the oleogel exhibited the lowest values for these parameters. Two groups were identified using hierarchical clustering analysis: one including ointments (Group 1) and the other including creams and an oleogel (Group 2). For details, please see [14]. The most important predictive factor was adhesiveness, followed by firmness. Ointments (Group 1) showed higher firmness, adhesiveness and consistency coefficient values compared to creams and gel (Group 2).

#### 2.2.5. Statistical Analysis

Descriptive analyses included absolute and relative frequencies for categorical variables and mean (standard deviation) or median (minimum–maximum) values for continuous variables, according to the symmetry of the distribution. The chi-squared (or Fisher) test was applied to investigate the independence of two factors; the Mann–Whitney test compared medians from two continuous variables.

The effect of the group formulations on the adherence/non-adherence to treatment, adjusted for potential confounders, was evaluated by multiple logistic regression. The statistical analyses were conducted using IBM SPSS Statistics 26.0. The significance level was fixed at 0.05.

## 3. Results

### 3.1. Sample Characterization

The sociodemographic and clinical characteristics of the sample are presented in Table 1.

### 3.2. Adherence to Topical Treatment

The mean treatment adherence was 65.4 ± 19.3%. The adherence rate results obtained using the self-report methodology (Adherence L: 70.0%) were much higher than the adherence rates obtained using the weighing methodology (Adherence W: 47.1%) (Table 2).

Adherence L represents the results for adherence calculated from the log, Adherence W represents the results for adherence calculated from the medication weight and Adherence Combo represents the results obtained from the combined methodology.

According to the dichotomized criterion for adherence to topical treatment, only 24.5% of the sample consisted of adherent participants. The majority of patients presented an adherence rate lower than 80%, but around 15% were over-adherent (Table 3).

**Figure 1:**
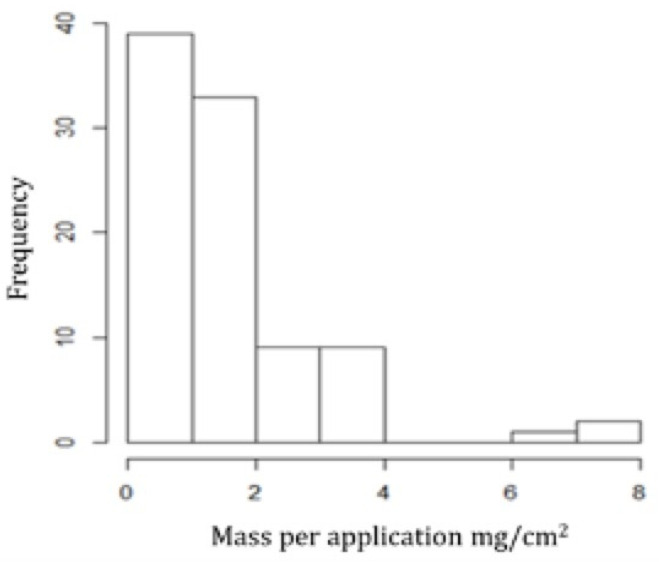
Histogram of the mass used per application.

The amount of medicine applied each time was quite variable, ranging from 0.07 to 7.8 mg/cm^2^ with a median value of 1.16 mg/cm^2^, thus defining a right-skewed distribution (Figure 1).

The mass used on each application (normalized by area) did not differ between males and females, nor between the different dosage forms (grouped into two categories) (Table 4). It was, however, significantly higher when the affected area was small.

Regarding treatment-related variables (Table 5), for most of the patients, the treatment had already been prescribed (continued treatment), and some of the medicines were identical to those previously prescribed. Most of the patients were instructed how to apply the medicines, although they were not given written information. Slightly more than half of the patients used skin care products as a complement to psoriasis treatment and the majority used these products without being advised to do so by the physician.

Patients were asked to report the reasons for non-adherence (Table 6). A high number of patients did not select any of the 25 reasons proposed. Among the reasons for non-adherence identified in this work, forgetfulness and interference with daily activities were the most cited. Other reasons reported were directly related to the vehicle characteristics, such as staining of clothes, the length of time required for application and difficulty of spreading. The only patient who mentioned the high cost of treatment as a reason for non-adherence used a compounded preparation. Stopping the treatment due to treatment efficacy might correspond to the physician’s instructions.

### 3.3. Influence of Treatment-Related Variables on Adherence

None of the treatment-related variables tested were shown to influence treatment adherence (Table 7).

### 3.4. Influence of the Vehicle on Adherence

The effect of the type of dosage form (vehicle) on the adherence/non-adherence to the topical treatment, adjusted for the effects of other studied variables, was evaluated by multiple logistic regression. The best model included the dosage form cluster group, the affected area and an interaction term (Table 8). The model was significantly better than the null model (*p* = 0.019) and its area under the ROC (Receiver Operating Characteristic) curve was estimated at 0.697, reflecting an average discrimination ability.

Different negative associations between the extent of lesions and adherence to treatment were estimated, depending on the vehicle. More precisely, patients with larger affected areas were less adherent irrespective of the formulation used. Moreover, for extensive affected areas, creams/gels were associated with significantly higher adherence to treatment than ointments (OR = 2.726).

## 4. Discussion

The mean value of adherence to psoriasis topical treatment was 65.4 ± 19%. Since the causes of non-adherence are multifactorial and are categorized into five main dimensions according to WHO, i.e., socioeconomic factors, health care and system-related factors, therapy-related factors, condition-related factors and patient-related factors, the adherence results are expected to vary substantially among patients. The self-reported adherence (Adherence L: 70%) was much higher than that obtained by weighing the medicines (Adherence W: 47.1%). This variability further reinforces the usefulness of the Adherence Combo approach used in this work and reported herein for the first time. Usually, self-reported methods overestimate adherence [8,9,23,34], and this was corroborated in this study. The observed mean adherence is low. Adherence rates higher than 80% are important to obtain optimal clinical outcomes. The adherence rates reported in the literature for topical treatment of psoriasis are very variable and influenced by the method used to evaluate adherence to treatment. It is thus difficult to establish a direct comparison with our findings. When considering a dichotomized criterion for adherence, a great majority of the participants were non-adherent to topical treatment (75.5%), a value which is strikingly low and raises awareness about the urgency of finding effective strategies to improve medication adherence in psoriasis patients. The reasons behind this behavior are elusive. More than 30% of patients were unable to identify the reasons for non-adherence from the set of 25 options. Each patient has their own perception and beliefs about the disease and the treatment and might not even be aware of their contribution to their adherence behavior. It is thus extremely difficult to recognize the general reasons why patients fail to follow medical prescriptions. A qualitative methodology could add an in-depth understanding of the underlying reasons, beliefs, attitudes and motivations that govern such behavior [35]. Among the reasons for non-adherence identified in this work, forgetfulness and interference with daily activities were the most frequently cited. About 18% of patients did not apply the treatment because they forgot—a reason also previously described for psoriasis patients [36]. Forgetfulness is a common reason for non-adherence to any type of treatment [37]. Around one fifth of the patients (20.9%) who described the reasons for non-adherence (a total of 67 patients) reported that the treatment was effective. This justification for interrupting the treatment cannot be considered a meaningful reason for non-adherence, since it might have corresponded to the physician’s instructions (apply until remission of lesions is observed). Confirmation of this instruction is limited by the fact that most patients (74.3%) did not receive instructions about the treatment. Ineffective treatment as a reason for non-adherence was reported by about 12% of patients. Fouéré et al. 2005 and Brown et al. 2006 [38,39] reported that this was one of the main reasons for non-adherence to topical treatment in psoriasis patients. The lack of time to administer the treatment was reported by 25.4% of patients with psoriasis, according to Gokdemir et al. 2008 [40], and was also reported as relevant in the study conducted by Zaghlou et al. 2004 [36]. This explanation was also mentioned in this study, since “treatment interferes with daily activities” was reported by 19.4% of patients. Other reasons reported are directly related to the vehicle characteristics, such as staining of clothes (4.5%), the length of time required for application (6%) and difficulty of spreading (3%). This emphasizes the influence of the vehicle on adherence. Since ointments present high firmness and consistency coefficients, it is expected that they will take more time and effort to apply than the other test formulations. In addition, ointments used for psoriasis contain petrolatum and thus are greasy and can stain clothes. Furthermore, in accordance with our previous results, a sticky feeling is one of the parameters associated with lower patient satisfaction [14] and has previously been cited as one of the reasons for low adherence to topical treatment of psoriasis [41]. Gels and creams present much lower adhesiveness, firmness and consistency than ointments [14] and most of them have an aqueous external phase. All the aforementioned characteristics can help to explain the higher adherence found in patients with an extensive body area affected by lesions, who used gels and creams. These findings are of value to pharmaceutical industries in selecting the vehicle composition of topical drug products for psoriasis. It is noteworthy that the effectiveness of the drug product is also influenced by the excipients of the vehicle, because they influence the skin drug delivery. Thus, insights into both mechanical features and drug delivery should be taken into account in drug product design.

Regarding the amount of medicine applied, a high variability in the results was observed (Figure 1), perhaps reflecting patients’ ignorance about the dose to be used, an error in weighing the medicine package or even the non-return of all medicine packages used during treatment. In fact, all patients in this study were unaware of the dose to be administered, leaving this item in QATOP unanswered. Analyzing the median value and comparing this with the theoretical values reported in the literature shows that approximately half of patients administered less than the required amount of topical medicine (roughly 1.2 mg/cm^2^) [31,33]. This is a critical issue since the administration of the correct dose is essential for optimal clinical outcome in topical treatment. It is noteworthy that a higher mass was used when small areas were involved (Table 4). This may be related to difficulty in establishing the correct dose to be applied [42] and controlling the amount removed from the tube/recipient. In view of these findings, a topical dosing applicator that allows a dose to be dispensed with greater accuracy and precision [43] and an adequate methodology to measure the affected area [44] may contribute to better clarification of dosing instructions to patients.

## 5. Conclusions

Analyzing our results from the point of view of adherence to treatment, we can conclude that gels and creams (Group 2) may offer greater ease of application, since they have lower firmness, consistency and adhesiveness compared to ointments (Group 1). This may justify the comparatively increased adherence observed with these dosage forms when the affected area is extensive. To improve adherence to topical treatment, and considering our results, the following recommendations to health professionals dealing with psoriasis patients can be suggested: (i) Patients with a greater affected body area are at high risk of failing to comply with the prescribed treatment and should be recipients of tailored interventions. (ii) The selection of the treatment should also consider the pharmaceutical dosage form, besides following clinical guidance, especially in the case of patients with greater affected body areas. After considering the best treatment options according to clinical guidance and available data on the influence of the vehicle on effectiveness, it could be of value to allow the patients to try vehicle samples and select the most suitable dosage form. (iii) Given patients’ ignorance of the dose of medication to be administered and the low frequency of patients who received written information about dosage (dose, frequency of administration and duration of treatment), it would be useful for the medical prescription to be accompanied by information on the mode of administration. Considering the complexity of adherence measurement, and in particular in the context of topical treatments, the combined methodology herein adopted could be taken as the gold standard in future adherence studies, thus allowing a more comprehensive evaluation and a direct comparison of results. The insight obtained in this work also provides support and a reference for pharmaceutical industries in developing topical formulations that effectively address the public health problem of non-adherence.

## Figures and Tables

**Table 1:** Sociodemographic and clinical characteristics of the patients.

Sociodemographic/Clinical Variable	Categories	*N*	%
Gender	Male	57	55.9
	Female	45	44.1
Marital status	Single/divorced/widowed	32	31.4
	Married	70	68.6
Education	Secondary/primary	88	87.2
	Higher education	13	12.8
Employment status	Employed/student	62	61.4
	Unemployed/retired	39	38.6
Family history of psoriasis	Yes	54	54.0
	No	46	46.0
Comorbidities	Yes	56	54.9
	No	46	45.1
Severity of psoriasis (SAPASI)	Mild	36	35.6
	Moderate	52	51.5
	Severe	11	10.9

**Table 2:** Mean values (and standard deviations) of adherence to topical treatment.

Adherence Measure	*N*	Mean (%)	SD
Adherence L	95	70.0	25.9
Adherence W	88	47.1	28.2
Adherence Combo	90	65.4	19.3

**Table 3:** Numbers and percentages of patients in each adherence level.

Adherence Rate	*N*	(%)
Less than 80%	57	60.6
Between 80 and 120%	23	24.5
More than 120%	14	14.9

**Table 4:** Mass used per application (mg/cm^2^).

Variable	Categories	Mass Per Application (mg/cm^2^)			
		Median	Minimum	Maximum	*p*-Value
Clusters	Group 1	1.17	0.11	3.30	0.744
	Group 2	1.11	0.07	7.61	
Gender	Male	1.19	0.18	7.61	0.720
	Female	1.14	0.07	7.98	
Affected area	Small	1.49			0.002
	Extensive	0.76			

**Table 5:** Treatment-related variables for the sample.

Treatment Related Variables	Categories	*N*	%
It is the first time this treatment has been prescribed?	Yes	35	36.1
	No	62	63.9
Some medications are identical to the ones you were taking?	Yes	67	81.7
	No	15	18.3
You were instructed on how to apply the medication?	Yes	87	94.6
	No	5	5.4
Information about the treatment was provided?	Yes	26	25.7
	No	75	74.3
The use of skin care products was recommended by the physician?	Yes	36	38.3
	No	58	61.7
Was clarification provided about the treatment at the pharmacy?	Yes	3	3.1
	No	93	96.9
Was the medicine applied only by you?	Yes	76	76.8
	No	23	23.2
Did you use skin care products as a complement to psoriasis treatment?	Yes	58	62.4
	No	35	37.6

**Table 6:** Reasons for non-adherence reported by the patients.

Reasons for Non-Adherence	*N* (Total 67)	%
Medication was effective	14	20.9
Treatment interferes with activities	13	19.4
Forgetfulness	12	17.9
Medicine was not effective	8	11.9
Difficult to access lesion’s anatomic site	4	6.0
Long application time	4	6.0
Fear of the side effects	3	4.5
Medicine leaves stains	3	4.5
Inconvenient time	2	3.0
Medicine is difficult to spread	2	3.0
Expensive treatment	1	1.5
Medicine caused itching	1	1.5
Patients that did not identify any reason	35	34.3
	***N*** **(Total 102)**	

**Table 7:** Association between adherence and treatment-related variables.

Treatment Related Variables	Categories	Adherence No		Adherence Yes		*p*-Value
		N	%	N	%	
Dosage form group	Group 1	21	32.3	10	45.5	0.195
	Group 2	44	67.7	12	54.5	
First-time prescribed treatment	No	41	60.3	15	71.4	0.506
	Yes	27	39.7	6	28.6	
Mode of application of medication was instructed	No	3	4.5	1	5.6	1
	Yes	64	95.5	17	94.4	
Treatment information was provided	No	54	77.1	14	60.9	0.209
	Yes	16	22.9	9	39.1	
Skin care products were recommended	No	42	64.6	13	61.9	1
	Yes	23	35.4	8	38.1	
Skin care products were used	No	30	48.4	10	45.5	1
	Yes	32	51.6	12	54.5	

**Table 8:** Results of the logistic regression model for the influence of the vehicle on adherence.

Variables	Coefficient	Standard Error	*p*-Value	95% CI
Constant	0.236	0.503	0.627	−0.721; 1.231
Group 2	−1.460	0.657	0.020	−2.765; −0.227
Extensive area	−2.505	1.019	0.003	−4.874; −0.781
Group 2 * Extensive area	2.463	1.208	0.025	0.296; 5.086

The reference classes for the binary factors in the linear predictor are Group 1 (ointments) and small affected area.

## Data Availability

The data presented in this study are available on request from the corresponding author. The data are not publicly available due to privacy.

## References

[1] Almeida V., Leite A., Constante D., Correia R., Almeida I.F., Teixeira M., Vidal D.G., Pedrosa e Sousa H.F., Dinis M.A.P., Teixeira A. (2020). The mediator role of body image related cognitive fusion in the relation between disease severity perception, acceptance and psoriasis disability. Behav. Sci..

[2] Almeida V., Constante D., Leite A., Almeida I.F., Rocha J.C., Sá R., Teixeira M., Teixeira A. (2021). Influence of disease phase on embitterment and emotional dysregulation in psoriatic patients. Psychol. Health Med..

[3] Christophers E. (2001). Psoriasis—Epidemiology and clinical spectrum. Clin. Exp. Dermatol..

[4] Rendon A., Schäkel K. (2019). Psoriasis Pathogenesis and Treatment. Int. J. Mol. Sci..

[5] Okwundu N., Cardwell L., Cline A., Richardson I., Feldman S.R. (2021). Is topical treatment effective for psoriasis in patients who failed topical treatment?. J. Dermatol. Treat..

[6] Svendsen M.T., Jeyabalan J., Andersen K.E., Andersen F., Johannessen H. (2017). Worldwide utilization of topical remedies in treatment of psoriasis: A systematic review. J. Derm. Treat..

[7] Kuehl B., Shear N. (2018). The Evolution of Topical Formulations in Psoriasis. Ski. Ther. Lett..

[8] Bewley A., Page B. (2011). Maximizing patient adherence for optimal outcomes in psoriasis. J. Eur. Acad. Derm. Venereol..

[9] Belinchón I., Rivera R., Blanch C., Comellas M., Lizán L. (2016). Adherence, satisfaction and preferences for treatment in patients with psoriasis in the European Union: A systematic review of the literature. Patient Prefer. Adherence.

[10] Zschocke I., Mrowietz U., Karakasili E., Reich K. (2014). Non-adherence and measures to improve adherence in the topical treatment of psoriasis. J. Eur. Acad. Derm. Venereol..

[11] European Network to Advance Best Practices & Technology on Medication Adherence 2020. https://www.cost.eu/cost-action/european-network-to-advance-best-practices-technology-on-medication-adherence.

[12] Cutler R.L., Fernandez-Llimos F., Frommer M., Benrimoj C., Garcia-Cardenas V. (2018). Economic impact of medication nonadherence by disease groups: A systematic review. BMJ Open.

[13] Council of Europe (2019). European Pharmacopoeia.

[14] Teixeira A., Vasconcelos V., Teixeira M., Almeida V., Azevedo R., Torres T., Sousa Lobo J.M., Costa P.C., Almeida I.F. (2019). Mechanical properties of topical anti-psoriatic medicines: Implications for patient satisfaction with treatment. AAPS PharmSciTech.

[15] Vasconcelos V., Teixeira A., Almeida V., Teixeira M., Ramos S., Torres T., Sousa Lobo J.M., Almeida I.F. (2018). Patient preferences for attributes of topical anti-psoriatic medicines. J. Dermatol. Treat..

[16] Lambert J., Hol C.W., Vink J. (2015). Real-life effectiveness of once-daily calcipotriol and betamethasone dipropionate gel vs. ointment formulations in psoriasis vulgaris: Final analysis of the 52-week PRO-long study. J. Eur. Acad. Dermatol. Venereol..

[17] Paul C., Stein G.L., Cambazard F., Kalb R.E., Lowson D., Bang B., Griffiths C.E. (2017). Calcipotriol plus betamethasone dipropionate aerosol foam provides superior efficacy vs. gel in patients with psoriasis vulgaris: Randomized, controlled PSO-ABLE study. J. Eur. Acad. Derm. Venereol..

[18] Puig L., Carrascosa J.M., Belinchón I., Fernández-Redondo V., Carretero G., Ruiz-Carrascosa J.C., Careaga J.M., de la Cueva P., Gárate M.T., Ribera M. (2013). Adherence and Patient Satisfaction with Topical Treatment in Psoriasis, and the Use, and Organoleptic Properties of Such Treatments: A Delphi Study with an Expert Panel and Members of the Psoriasis Group of the Spanish Academy of Dermatology and Venereology. Actas Dermosifiliogr..

[19] Reich K., Bewley A. (2011). What is new in topical therapy for psoriasis?. J. Eur. Acad. Dermatol. Venereol..

[20] Iversen L., Jakobsen H.B. (2016). Patient Preferences for Topical Psoriasis Treatments are Diverse and Difficult to Predict. Dermatol. Ther. (Heidelb).

[21] Sandoval L.F., Huang K.E., Harrison J., Clark A., Feldman S.R. (2014). Calcipotriene 0.005%—Betamethasone dipropionate 0.064% ointment versus topical suspension in the treatment of plaque psoriasis: A randomized pilot study of patient preference. Cutis.

[22] Van de Kerkhof P.C., Franssen M., de La Brassine M., Kuipers M. (2001). Calcipotriol cream in the morning and ointment in the evening: A novel regimen to improve compliance. J. Dermatol. Treat..

[23] Teixeira A., Teixeira M., Almeida V., Torres T., Sousa Lobo J.M., Almeida I.F. (2016). Methodologies for medication adherence evaluation: Focus on psoriasis topical treatment. J. Derm. Sci..

[24] Teixeira A., Teixeira M., Almeida V., Almeida I.F. (2017). Adherence to topical treatment in psoriasis. Adherence to Medical Plans for an Active and Healthy Ageing.

[25] Ribeiro C., Pereira A., Martins S., Teixeira A., Almeida V. Severidade da psoríase: Um estudo de validação do Índice Autoadministrado da Área e Severidade da Psoríase. Proceedings of the V Congresso Internacional de Psicologia Clínica.

[26] Feldman S.R., Fleischer A.B., Reboussin D.M., Rapp S.R., Lyn Exum M., Clark A.R., Nurre L. (1996). The self-administered psoriasis area and severity index is valid and reliable. J. Investig. Dermatol..

[27] Fleischer A.B., Rapp S.R., Reboussin D.M., Vanarthos J.C., Feldman S.R. (1994). Patient measurement of psoriasis disease severity with a structured instrument. J. Investig. Dermatol..

[28] Henseler T., Schmitt-Rau K. (2008). A comparison between BSA, PASI, PLASI and SAPASI as measures of disease severity and improvement by therapy in patients with psoriasis. Int. J. Derm..

[29] Sampogna F., Sera F., Mazzotti E., Pasquini P., Picardi A., Abeni D., IDI Multipurpose Psoriasis Research on Vital Experiences (IMPROVE) Study Group (2003). Performance of the self-administered Psoriasis Area and Severity Index in evaluating clinical and sociodemographic subgroups of patients with psoriasis. Arch. Dermatol..

[30] Schlagel C.A., Sanborn E.C. (1964). The Weights of Topical Preparations Required for Total and Partial Body Inunction. J. Investig. Dermatol..

[31] Long C.C., Finlay A.Y. (1991). The finger-tip unit—a new practical measure. Clin. Exp. Dermatol..

[32] Long C.C., Finlay A.Y., Averill R.W. (1992). The Rule of Hand: 4 Hand Areas=2 FTU=1 g. Arch. Dermatol..

[33] Teixeira A., Oliveira C., Teixeira M., Gaio A.R., Sousa Lobo J.M., de Almeida I.F.M., Almeida V. (2017). Development and Validation of a Novel Questionnaire for Adherence with Topical Treatments in Psoriasis (QATOP). Am. J. Clin. Dermatol..

[34] Devaux S., Castela A., Archier E., Gallini A., Joly P., Misery L., Aractingi S., Aubin F., Bachelez H., Cribier B. (2012). Adherence to topical treatment in psoriasis: A systematic literature review. J. Eur. Acad. Derm. Venereol..

[35] Kaae S., Traulsen J.M., Babar Z.-U.-D. (2015). Qualitative methods in pharmacy practice research. Pharmacy Practice Research Methods.

[36] Zaghloul S.S., Goodfield M.J.D. (2004). Objective Assessment of Compliance with Psoriasis Treatment. Arch. Dermatol..

[37] Brown M.T., Sinsky C.A. (2013). Medication Adherence: We Didn’t Ask and They Didn’t Tell. Fam. Pract. Manag..

[38] Fouéré S., Adjadj L., Pawin H. (2005). How patients experience psoriasis: Results from a European survey. J. Eur. Acad. Derm. Venereol..

[39] Brown K.K., Rehmus W.E., Kimball A.B. (2006). Determining the relative importance of patient motivations for nonadherence to topical corticosteroid therapy in psoriasis. J. Am. Acad. Dermatol..

[40] Gokdemir G., Arı S., Köşlü A. (2008). Adherence to treatment in patients with psoriasis vulgaris: Turkish experience. J. Eur. Acad. Dermatol. Venereol..

[41] Park E.K., Song K.W. (2010). Rheological evaluation of petroleum jelly as a base material in ointment and cream formulations: Steady shear flow behavior. Arch. Pharm. Res..

[42] Teixeira A., Teixeira M., Herdeiro M., Vasconcelos V., Correia R., Bahia M., Almeida I., Vidal D., Sousa H., Dinis M. (2021). Knowledge and Practices of Community Pharmacists in Topical Dermatological Treatments. Int. J. Environ. Res. Public Health.

[43] Rasmussen G., Bech L.L., Nielsen T.W. (2015). An Applicator Delivery System for Fixed-Combination Calcipotriene Plus Betamethasone Dipropionate Topical Suspension (Gel): Innovating Psoriasis Vulgaris Treatment through Patient Collaboration. Dermatol. Ther. (Heidelb).

[44] Sobral P., Teixeira A., Almeida I.F., Almeida V., Rocha Á., Adeli H., Dzemyda G., Moreira F., Ramalho Correia A.M. (2021). Mobile System for Personal Support to Psoriatic Patients. Trends and Applications in Information Systems and Technologies, Proceedings of the WorldCIST: World Conference on Information Systems and Technologies (WorldCIST 2021), Terceira Island, Portugal, 30 March–2 April 2021.

